# Information Generating Function of Ranked Set Samples

**DOI:** 10.3390/e23111381

**Published:** 2021-10-21

**Authors:** Omid Kharazmi, Mostafa Tamandi, Narayanaswamy Balakrishnan

**Affiliations:** 1 Department of Statistics, Faculty of Mathematical Sciences, Vali-e-Asr University of Rafsanjan, Rafsanjan P.O. Box 518, Iran; omid.kharazmi@vru.ac.ir (O.K.); tamandi@vru.ac.ir (M.T.); 2 Department of Mathematics and Statistics, McMaster University, Hamilton, ON L8S 4L8, Canada

**Keywords:** information generating function, relative information generating function, Kullback–Leibler divergence, ranked set sampling, simple random sampling

## Abstract

In the present paper, we study the information generating (IG) function and relative information generating (RIG) function measures associated with maximum and minimum ranked set sampling (RSS) schemes with unequal sizes. We also examine the IG measures for simple random sampling (SRS) and provide some comparison results between SRS and RSS procedures in terms of dispersive stochastic ordering. Finally, we discuss the RIG divergence measure between SRS and RSS frameworks.

## 1. Introduction

Moment generating function (MGF) plays an important role in statistical distribution theory. Its derivatives evaluated at zero yield the moments of the considered distribution. Information generating (IG) functions have also been used in information theory, in addition to the moment generating function, to generate some well-known information measures such as Shannon entropy and Kullback–Leibler divergence.

The IG function of a probability model *f* was first introduced by Golomb [1], whose first derivative evaluated at one provides Shannon entropy for that probability model.

Suppose the variable *X* has an absolutely continuous probability density function (PDF) *f*. Then, the IG function of density f, for any α>0, is defined as

(1) $$\begin{array}{c} G_{\alpha }\left(X\right)=\int_{X}f^{\alpha }\left(x\right)dx, \end{array}$$

when the integral is finite. In order to simplify the notation, we do not use X in the integration with respect to dx throughout the article, unless a distinction needs to be made. The following properties of $G_{\alpha }\left(X\right)$ in (1) have been stated in Golomb [1]:

(2) $$\;\;\;\;\;\;\;\left(i\right)\;\;\;\;\;\;G_{1}\left(X\right)=1;\;\;\;\;\;\;\;\;\;\;\;\;\left(ii\right)\;\;\;\;\;\frac{\partial }{\partial \alpha }G_{\alpha }{\left(X\right)|}_{\alpha =1}=-H\left(X\right),$$

where H(X) is the Shannon entropy defined as H(X)=−∫f(x)logf(x)dx. In particular, when α=2, the IG measure is simply $\int_{X}f^{2}\left(x\right)dx$, known as informational energy (IE) function. The IG function and its extensions have been used extensively in chemistry and physics to discuss the atomic structure of a given phenomena or system; for more details, one may see López-Ruiz et al. [2]. In addition, the IG function, known as entropic moment in chemistry and physics literature, plays a key role in chaos theory and non-extensive thermodynamics. Note that the IG function is closely linked to Tsallis and Rényi entropies. The entropic moment measure, as well as the information entropy, reflect on the degree of spread of a probabilistic model, see Bercher [3].

Recently, Clark [4] has presented an analogous IG function for stochastic processes to assist in the derivation of information measures for point processes.

Guiasu and Reischer [5] proposed relative information generating (RIG) function between two density functions, whose first derivative evaluated at 1, yields Kullback–Leibler (KL) divergence (Kullback and Leibler, [6]) measure.

Suppose the variables *X* and *Y* have absolutely continuous density functions *f* and *g*, respectively. Then, the RIG function, for any α>0, is defined as

(3) $$\begin{array}{c} R_{\alpha }\left(X,Y\right)=\int g\left(x\right){\left\{\frac{f(x)}{g(x)}\right\}}^{\alpha }dx \end{array}$$

when the integral is finite. The KL divergence is then obtained, from its first derivative, as

(4) $$KL\left(X,Y\right)=\frac{\partial }{\partial \alpha }R_{\alpha }{\left(X,Y\right)|}_{\alpha =1}=\int f\left(x\right)\left(\log\frac{f(x)}{g(x)}\right)dx.$$

One may refer to Clark [4] and Mares et al. [7] for some discussions on the usefulness and applications of the RIG function

The main objective of this paper is to study the IG and RIG information measures associated with ranked set sampling (RSS) schemes. The analysis of information content in various sampling strategies is of great importance in sampling theory. In this regard, information theory provides specifically a framework for the quantification of information content in a given source with a probabilistic structure under different sampling strategies. Among various strategies discussed in sampling theory, we focus here on some well-known strategies that are known to be efficient. A cost-effective survey sampling method, known as ranked set sampling (RSS), was first introduced by McIntyre [8]. He specifically introduced RSS to estimate the mean of a population based on a given simple random sample (SRS) of size *n* and observed that the estimator based on RSS is an unbiased estimator with a smaller variance as compared to the mean of a SRS. The RSS and some of its generalizations have been discussed rather extensively in the literature. For example, Frey [9]; Park and Lim [10]; and Chen, Bai, and Sinha [11] have all discussed the information content in RSS based on Fisher entropy, while Tahmasebi et al. [12] have studied the Tsallis entropy based on maximum RSS scheme. Therefore, considering the importance of this issue and the connection between information theory and ranked set sampling theory, a systematic study of the IG function as generator function of some well-known information measures, in the framework of RSS strategy, seems to be necessary. This forms the primary motivation for the present study.

We now briefly introduce SRS and RSS strategies that will be used in the sequel. Let *X* be an absolutely continuous random variable with PDF *f*. Then, a SRS of size *n*, derived from the random variable *X*, is denoted by $X_{SRS}=\left\{X_{i},\;i=1,\ldots ,n\right\}.$ Further, suppose a random sample of size $n^{2}$ is selected and is randomly divided into *n* groups of equal size *n*. Then, a one-cycle RSS is observed in the following manner:

$$\begin{array}{cccccccccccccc} \; & 1: & \; & \underline{X_{(1:n)1}} & \; & X_{(2:n)1} & \; & \cdots & \; & X_{(n:n)1} & \; & \to & \; & X_{\left(1:n\right)}=X_{(1:n)1}\\ \; & 2: & \; & X_{(1:n)2} & \; & \underline{X_{(2:n)2}} & \; & \cdots & \; & X_{(n:n)2} & \; & \to & \; & X_{\left(2:n\right)}=X_{(2:n)2}\\ \; & \;\;\vdots & \; & \;\;\;\vdots & \; & \;\;\;\vdots & \; & \;\ddots & \; & \;\;\;\vdots & \; & \;\;\;\vdots & \; & \;\;\;\;\;\;\;\;\;\vdots \\ \; & n: & \; & X_{(1:n)n} & \; & X_{(2:n)n} & \; & \cdots & \; & \underline{X_{(n:n)n}} & \; & \to & \; & X_{\left(n:n\right)}=X_{(n:n)n}. \end{array}$$

As we see from the above representation, the recorded sample in each group of SRS with size *n* corresponds to the *i*th order statistic. Thus, the RSS vector of observations is given by $X_{RSS}^{\left(n\right)}=\left\{X_{i:n},\;i=1,\cdots n\right\}$, where $X_{i:n}$ is the *i*th order statistic based on a given SRS of size *n* with PDF *f* and cumulative distribution function (CDF) *F*. Then, the PDF of $X_{i:n}$ is known to be

(5) $$f_{i:n}\left(x\right)=\frac{n!}{(i-1)!(n-i)!}f\left(x\right)F^{i-1}\left(x\right){\left(1-F\left(x\right)\right)}^{n-i}.$$

Here, $X_{i:n}$ corresponds to the *i*th order statistic, and with that taking the value *x*, there will be i−1 observations less than *x* each with probability F(x) and n−i observations greater than *x* each with probability 1−F(x). For pertinent details, one may refer to the authoritative book on this subject by Arnold et al. [13].

Maximum and minimum ranked set sampling schemes are two useful modifications of ranked set sampling procedure. A maximum RSS is given by $X_{MRSS}^{\left(n\right)}=\left\{X_{(i)i},\;i=1,\cdots ,n\right\}$, where $X_{(i)i}$ is the largest order statistic based on a SRS of size *i* from *f*. Similarly, a minimum RSS is given by $X_{mRSS}^{\left(n\right)}=\left\{X_{(1)i},\;i=1,\cdots ,n\right\}$, where $X_{(1)i}$ is the smallest order statistic based on a SRS of size *i* from *f*. From (5), the PDF of $X_{(1)i}$ is given by

(6) $$f_{(1)i}\left(x\right)=i{\left[\bar{F}\left(x\right)\right]}^{i-1}f\left(x\right),\;i=1,\ldots ,n,$$

where $\bar{F}=1-F,$ is the survival function of *X*. Similarly, the PDF of $X_{(i)i}$ is given by

(7) $$f_{(i)i}\left(x\right)=i{\left[F(x)\right]}^{i-1}f\left(x\right),\;i=1,\ldots ,n.$$

The corresponding CDFs of (6) and (7) are given by $1-{\bar{F}}^{i}\left(x\right)$ and $F^{i}\left(x\right)$, respectively.

The purpose of this work is twofold. The first part is to derive IG measures for the SRS and RSS, and especially in maximum and minimum RSS frameworks, and provide some comparison results associated with IG measures of these observations based on dispersive stochastic ordering. In the second part, we further study the RIG divergence measure between SRS and RSS, and specifically the RIG divergence measure between minimum and maximum RSS procedures.

The rest of this paper is organized as follows. In Section 2, we consider the information generating function and establish some results for SRS and RSS procedures. We show that the IG measures of SRS and RSS can be expressed based on different orders of fractional Shannon entropy. Moreover, we examine the monotonicity properties of IG measure for vectors $X_{MRSS}^{\left(n\right)}$ and $X_{mRSS}^{\left(n\right)}$ based on a sample of size *n*, under a mild condition. In Section 3, we discuss the comparison of information generating functions for SRS and RSS frameworks in terms of dispersive stochastic ordering. Next, in Section 4, we study the RIG measures for vectors $X_{SRS}^{\left(n\right)}$, $X_{MRSS}^{\left(n\right)}$ and $X_{MRSS}^{\left(n\right)}$. Finally, we make some concluding remarks in Section 5.

## 2. IG Measures Based on SRS and RSS Schemes

In this section, we first consider the IG measure for SRS and then for RSS schemes. Specifically, we discuss the IG measure for the maximum and minimum RSS schemes.

### 2.1. IG Measure Based on SRS Scheme

Let $X_{SRS}^{\left(n\right)}=\left(X_{1},\cdots ,X_{n}\right)$ be a SRS of size *n* obtained from PDF *f*. Then, the IG measure of vector $X_{SRS}^{\left(n\right)}$ is given by

(8) $$\begin{array}{ccc} G_{\alpha }\left(X_{SRS}^{\left(n\right)}\right) & = & \int \cdots \int f^{\alpha }\left(x_{1}\right)\cdots f^{\alpha }\left(x_{n}\right)dx_{1}\cdots dx_{n}\\ & = & \prod_{i=1}^{n}\int f^{\alpha }\left(x_{i}\right)dx_{i}={\left(\int f^{\alpha }\left(x\right)dx\right)}^{n}={\left(G_{\alpha }\left(X\right)\right)}^{n}. \end{array}$$

**Lemma** **1.** *Suppose the random variable X has density function f. Then, we have*

$$G_{\alpha }\left(X_{SRS}^{\left(n\right)}\right)={\left\{\sum_{j=0}^{\infty }\frac{{\left(1-\alpha \right)}^{j}}{j!}H_{j}\left(f\right)\right\}}^{n},$$

*where $H_{j}\left(f\right)$ is the extended fractional Shannon entropy of order n defined as $H_{j}\left(f\right)=\int {\left\{-\log f(x)\right\}}^{j}f\left(x\right)dx.$ For more details about fractional Shannon entropy, one may refer to Xiong et al. [14].*

**Proof.**  From the definition of IG measure of $X_{SRS}^{\left(n\right)}$ in (8) and using Lemma 1 of Kharazmi and Balakrishnan [15], we have

$$\begin{array}{ccc} {\left\{G_{\alpha }\left(X_{SRS}^{\left(n\right)}\right)\right\}}^{\frac{1}{n}} & = & E\left[e^{\left(\alpha -1)\log f(X\right)}\right]=\sum_{j=0}^{\infty }\frac{{\left(1-\alpha \right)}^{j}}{j!}\int {\left\{-\log f(x)\right\}}^{j}f\left(x\right)dx\\ & = & \sum_{j=0}^{\infty }\frac{{\left(1-\alpha \right)}^{j}}{j!}H_{j}\left(f\right), \end{array}$$

as required. □

### 2.2. IG Measure Based on RSS Scheme

Suppose $X_{1},\ldots ,X_{n}$ are independent and identically distributed (iid) variables from an absolutely continuous CDF *F* and PDF *f*, and $X_{1:n},\ldots ,X_{n:n}$ are the corresponding order statistics. We then present the IG measure of vector $X_{RSS}^{\left(n\right)}=\left\{X_{i:n},\;i=1,\cdots n\right\}$ in the following theorem.

**Theorem** **1.** *Let $X_{RSS}^{\left(n\right)}$ denote a RSS from density function f. Then, the IG measure of vector $X_{RSS}^{\left(n\right)}$, for α>0, is given by*

(9) $$\begin{array}{c} G_{\alpha }\left(X_{RSS}^{\left(n\right)}\right)=\prod_{i=1}^{n}G_{\alpha }\left(X_{i:n}\right)=\psi \left(\alpha ,n\right)\prod_{i=1}^{n}E\left[f^{\alpha -1}\left(F^{-1}\left(V_{i}\right)\right)\right], \end{array}$$

*where $\psi \left(\alpha ,n\right)=\prod_{i=1}^{n}\frac{B\left(\alpha (i-1)+1,\alpha (n-i)+1\right)}{B^{\alpha }\left(i,n-i+1\right)}$, and $V_{i}$ has $Beta\left(\alpha (i-1)+1,\alpha (n-i)+1\right)$ distribution with PDF*

$$f_{V_{i}}\left(v\right)=\frac{1}{B\left(\alpha (i-1)+1,\alpha (n-i)+1\right)}v^{\alpha (i-1)}{\left(1-v\right)}^{\alpha (n-i)},\;0<v<1.$$

**Proof.**  From the definition of IG measure in (1) for vector $X_{RSS}^{\left(n\right)}$ and setting v=F(x), we have

$$\begin{array}{ccc} G_{\alpha }\left(X_{RSS}^{\left(n\right)}\right) & = & \prod_{i=1}^{n}G_{\alpha }\left(X_{i:n}\right)=\prod_{i=1}^{n}\int f_{i:n}^{\alpha }\left(x\right)dx\\ & = & \prod_{i=1}^{n}\int \frac{1}{B^{\alpha }\left(i,n-i+1\right)}f^{\alpha }\left(x\right){\left[F(x)\right]}^{\alpha (i-1)}{\left[1-F(x)\right]}^{\alpha (n-i)}dx\\ & = & \psi \left(\alpha ,n\right)\prod_{i=1}^{n}E\left[f^{\alpha -1}\left(F^{-1}\left(V_{i}\right)\right)\right], \end{array}$$

as required. □

Based on the definition of fractional Shannon entropy and Lemma 1 of Kharazmi and Balakrishnan [15], we can present an alternative representation for $G_{\alpha }\left(X_{RSS}^{\left(n\right)}\right)$ as

$$\begin{array}{ccc} G_{\alpha }\left(X_{RSS}^{\left(n\right)}\right) & = & \sum_{j=0}^{\infty }\frac{{\left(1-\alpha \right)}^{j}}{j!}\prod_{i=1}^{n}H_{j}\left(f_{i:n}\right), \end{array}$$

where $H_{j}$ is the fractional Shannon entropy of order *j* and $f_{i:n}$ is the PDF of $X_{i:n}$ as given in (5).

**Example** **1.** *Let X be an exponential variable with PDF $f\left(x\right)=\lambda e^{-\lambda x},\;\lambda >0,\;x>0$. From (1) and (8), we then find $G_{\alpha }\left(X_{SRS}^{\left(n\right)}\right)=\frac{\lambda^{n(\alpha -1)}}{\alpha^{n}}.$ On the other hand, as $f(F^{-1}\left(u\right))=\lambda \left(1-u\right),\;0<u<1,$ from (9), we find*

$$\begin{array}{c} G_{\alpha }\left(X_{RSS}^{\left(n\right)}\right)=\lambda^{n(\alpha -1)}\prod_{i=1}^{n}\frac{B\left(\alpha (i-1)+1,\alpha (n-i+1)\right)}{B^{\alpha }\left(i,n-i+1\right)}. \end{array}$$

Next, we discuss the IG measure for maximum and minimum RSS schemes with vectors $X_{MRSS}^{\left(n\right)}=\left\{X_{(i)i},\;i=1,\cdots n\right\}$ and $X_{mRSS}^{\left(n\right)}=\left\{X_{(1)i},\;i=1,\cdots n\right\}$, respectively.

**Theorem** **2.** *Let $X_{mRSS}^{\left(n\right)}$ and $X_{MRSS}^{\left(n\right)}$ denote the minimum and maximum RSS schemes from density function f, respectively. Then, the IG measures of vectors $X_{mRSS}^{\left(n\right)}$ and $X_{MRSS}^{\left(n\right)}$, for α>0, are given by*

(10) $$\begin{array}{c} G_{\alpha }\left(X_{mRSS}^{\left(n\right)}\right)=\prod_{i=1}^{n}G_{\alpha }\left(X_{(1)i}\right)=c\left(\alpha ,n\right)\prod_{i=1}^{n}E\left\{f^{\alpha -1}\left(F^{-1}\left(U_{i}\right)\right)\right\} \end{array}$$

*and*

(11) $$\begin{array}{c} G_{\alpha }\left(X_{MRSS}^{\left(n\right)}\right)=\prod_{i=1}^{n}G_{\alpha }\left(X_{(i)i}\right)=c\left(\alpha ,n\right)\prod_{i=1}^{n}E\left\{f^{\alpha -1}\left(F^{-1}\left(V_{i}\right)\right)\right\}, \end{array}$$

*respectively, where $U_{i}$ has Beta(1,α(i−1)+1) and $V_{i}$ has Beta(α(i−1)+1,1) distributions, with $c\left(\alpha ,n\right)=\frac{{\left(n!\right)}^{\alpha }}{\prod_{i=1}^{n}\left(\alpha \left(i-1\right)+1\right)}.$*

**Proof.**  From the definition of IG measure in (1) and using the PDF of $X_{(1)i}$ in (6), upon setting u=F(x), we get

$$\begin{array}{ccc} G_{\alpha }\left(X_{mRSS}^{\left(n\right)}\right) & = & \prod_{i=1}^{n}\int_{-\infty }^{\infty }f_{(1)i}^{\alpha }\left(x\right)dx=\prod_{i=1}^{n}\int_{-\infty }^{\infty }i^{\alpha }{\left[\bar{F}\left(x\right)\right]}^{\alpha (i-1)}f^{\alpha }\left(x\right)dx\\ & = & {\left(n!\right)}^{\alpha }\prod_{i=1}^{n}\int_{0}^{1}{\left(1-u\right)}^{\alpha (i-1)}f^{\alpha -1}\left(F^{-1}\left(u\right)\right)du\\ & = & c\left(\alpha ,n\right)\prod_{i=1}^{n}E\left\{f^{\alpha -1}\left(F^{-1}\left(U_{i}\right)\right)\right\}, \end{array}$$

as required. The proof of (11) is similar, and is therefore omitted for the sake of brevity. □

**Example** **2.** *For the exponential PDF considered in Example 1, by using (10) and (11), we find*

 *(i)* *$G_{\alpha }\left(X_{mRSS}^{\left(n\right)}\right)=\frac{{\left(n!\right)}^{\alpha -1}\lambda^{n(\alpha -1)}}{\alpha^{n}}$,*
 *(ii)*
$G_{\alpha }\left(X_{MRSS}^{\left(n\right)}\right)=G_{\alpha }\left(X_{mRSS}^{\left(n\right)}\right)\left((\alpha -1)!\right)\prod_{i=1}^{n}\frac{\Gamma (\alpha (i-1)+1)}{\Gamma (\alpha i)}.$

**Figure 1:**
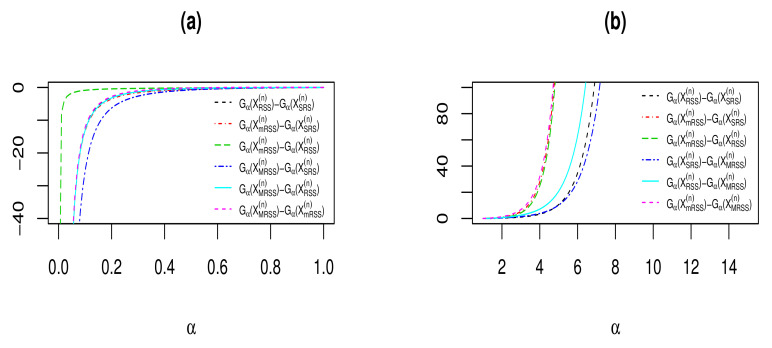
The differences between IG measures for exponential distribution with λ=2 and n=2 when 0<α<1 (**a**) and α>1 (**b**).

Figure 1 shows the differences between IG measures of vectors $X_{SRS}^{\left(n\right)}$, $X_{RSS}^{\left(n\right)}$, $X_{MRSS}^{\left(n\right)}$, and $X_{MRSS}^{\left(n\right)}$ in Examples 1 and 2, for different values of α>0 and n=2. From Figure 1, it is easy to observe that for α∈(0,1], the IG differences are negative and increasing (Panel (a)), while for α∈[1,∞), the IG differences are positive and increasing (Panel (b)).

Suppose *X* has CDF *F* and PDF *f*, and the vectors $X_{MRSS}^{\left(n\right)}$ and $X_{mRSS}^{\left(n\right)}$ are the associated maximum and minimum RSS schemes based on a sample of size *n*. Then, the following results present the monotonicity properties of IG measures for vectors $X_{MRSS}^{\left(n\right)}$ and $X_{mRSS}^{\left(n\right)}$.

**Theorem** **3.** *Consider the IG measure of vector $X_{MRSS}^{\left(n\right)}$. If $f(F^{-1}\left(u\right))\geq 1$ for all 0<u<1, then:*

 *(i)* *If α≥1, $G_{\alpha }\left(X_{MRSS}^{\left(n\right)}\right)$ is increasing in n;*
 *(ii)* *If α≤1, $G_{\alpha }\left(X_{MRSS}^{\left(n\right)}\right)$ is decreasing in n.*

**Proof.**  By using the assumption and the definition of IG measure for the vector $X_{MRSS}^{\left(n\right)}$ in (11), we have

$$\begin{array}{ccc} \frac{G_{\alpha }\left(X_{MRSS}^{\left(n+1\right)}\right)}{G_{\alpha }\left(X_{MRSS}^{\left(n\right)}\right)} & = & \frac{\prod_{i=1}^{n+1}\int_{-\infty }^{\infty }f_{(i)i}^{\alpha }\left(x\right)dx}{\prod_{i=1}^{n}\int_{-\infty }^{\infty }f_{(i)i}^{\alpha }\left(x\right)dx}=\int_{-\infty }^{\infty }f_{(n+1)n+1}^{\alpha }\left(x\right)dx\\ & = & {\left(n+1\right)}^{\alpha }\int_{0}^{1}u^{\alpha n}f^{\alpha -1}\left(F^{-1}\left(u\right)\right)du\\ & \geq & {\left(n+1\right)}^{\alpha }\int_{0}^{1}u^{\alpha n}du=\frac{{\left(n+1\right)}^{\alpha }}{\alpha n+1}\geq 1,\;\;for\;\;\alpha \geq 1, \end{array}$$

which proves Part (i). Part (ii) can be proved in an analogous manner. □

**Theorem** **4.** *Consider the IG measure of vector $X_{mRSS}^{\left(n\right)}$. If $f(F^{-1}\left(u\right))\geq 1$ for all 0<u<1, then:*

 *(i)* *If α≥1, $G_{\alpha }\left(X_{mRSS}^{\left(n\right)}\right)$ is increasing in n;*
 *(ii)* *If α≤1, $G_{\alpha }\left(X_{mRSS}^{\left(n\right)}\right)$ is decreasing in n.*

**Proof.**  By using the assumptions and the definition of IG measure for the vector $X_{mRSS}^{\left(n\right)}$ in (10), we have

$$\begin{array}{ccc} \frac{G_{\alpha }\left(X_{mRSS}^{\left(n+1\right)}\right)}{G_{\alpha }\left(X_{mRSS}^{\left(n\right)}\right)} & = & \frac{\prod_{i=1}^{n+1}\int_{-\infty }^{\infty }f_{(1)i}^{\alpha }\left(x\right)dx}{\prod_{i=1}^{n}\int_{-\infty }^{\infty }f_{(i1)i}^{\alpha }\left(x\right)dx}=\int_{-\infty }^{\infty }f_{(1)n+1}^{\alpha }\left(x\right)dx\\ & = & \int_{-\infty }^{\infty }{\left(n+1\right)}^{\alpha }{\left(1-F(x)\right)}^{\alpha n}f^{\alpha }\left(x\right)dx\\ & = & {\left(n+1\right)}^{\alpha }\int_{0}^{1}{\left(1-u\right)}^{\alpha n}f^{\alpha -1}\left(F^{-1}\left(u\right)\right)du\\ & \geq & {\left(n+1\right)}^{\alpha }\int_{0}^{1}{\left(1-u\right)}^{\alpha n}du=\frac{{\left(n+1\right)}^{\alpha }}{\alpha n+1}\geq 1,\;\;for\;\;\alpha \geq 1, \end{array}$$

which proves Part (i). Part (ii) can be proved in an analogous manner. □

Next, we compare the IG measure of vector $X_{SRS}^{\left(n\right)}$ with those of $X_{mRSS}^{\left(n\right)}$ and $X_{MRSS}^{\left(n\right)}$.

**Theorem** **5.** *Consider the IG measures $G_{\alpha }\left(X_{SRS}^{\left(n\right)}\right)$, $G_{\alpha }\left(X_{mRSS}^{\left(n\right)}\right)$ and $G_{\alpha }\left(X_{MRSS}^{\left(n\right)}\right)$. Then:*

 *(i)* *If α≥1, $G_{\alpha }\left(X_{mRSS}^{\left(n\right)}\right)\leq {\left(n!\right)}^{\alpha }G_{\alpha }\left(X_{SRS}^{\left(n\right)}\right)$;*
 *(ii)* *If α≥1, $G_{\alpha }\left(X_{MRSS}^{\left(n\right)}\right)\leq {\left(n!\right)}^{\alpha }G_{\alpha }\left(X_{SRS}^{\left(n\right)}\right)$.*

**Proof.**  By the definition of IG measures of vectors $X_{SRS}^{\left(n\right)}$ and $X_{mRSS}^{\left(n\right)}$, we find

$$\begin{array}{ccc} G_{\alpha }\left(X_{mRSS}^{\left(n\right)}\right) & = & {\left(n!\right)}^{\alpha }\prod_{i=1}^{n}\int_{0}^{1}{\left(1-u\right)}^{\alpha (i-1)}f^{\alpha -1}\left(F^{-1}\left(u\right)\right)du\\ & \leq & {\left(n!\right)}^{\alpha }\prod_{i=1}^{n}\int_{0}^{1}f^{\alpha -1}\left(F^{-1}\left(u\right)\right)du\\ & = & {\left(n!\right)}^{\alpha }{\left\{\int_{0}^{1}f^{\alpha -1}\left(F^{-1}\left(u\right)\right)du\right\}}^{n}\\ & = & {\left(n!\right)}^{\alpha }G_{\alpha }\left(X_{SRS}^{\left(n\right)}\right), \end{array}$$

which proves Part (i). Part (ii) can be proved in an analogous manner. □

## 3. IG Ordering Results Based on the RSS Scheme

An important criterion for comparing the dispersions (or variabilities) of two variables (or distributions) is dispersive ordering. Let the variables *X* and *Y* have CDFs *F* and *G* and PDFs *f* and *g*, respectively. Then, *X* said to be less dispersed than *Y* (denoted by $X\leq_{disp}Y$) if $g\left(G^{-1}\left(x\right)\right)\leq f\left(F^{-1}\left(x\right)\right)$ for all x∈(0,1); see, for instance, Shaked and Shanthikumar [16] for relevant details.

**Definition** **1.** Let *X* and *Y* be two variables with IG measures $G_{\alpha }\left(f\right)$ and $G_{\alpha }\left(g\right)$, respectively. Then, *X* is said to be less than *Y* in the sense of information generating function, denoted by $X\leq_{IG}Y$, if $G_{\alpha }\left(f\right)\leq G_{\alpha }\left(g\right)$.

**Lemma** **2.** *Suppose $X\leq_{disp}Y$. Then:*

 *(i)* *If α≤1, $X\leq_{IG}Y$;*
 *(ii)* *If α≥1, $Y\leq_{IG}X$.*

**Proof.**  See Kharazmi and Balakrishnan [15] for a detailed proof. □

Now, we present the following theorem about the IG ordering for RSS schemes.

**Theorem** **6.** *Let ${\left\{X_{i}\right\}}_{1}^{\infty }$ be a sequence of i.i.d. variables from a deceasing failure rate (DFR) distribution. Then:*

 *(i)* *If α≤1, $X_{mRSS}^{\left(n\right)}\leq_{IG}X_{RSS}^{\left(n\right)}\leq_{IG}X_{MRSS}^{\left(n\right)}$;*
 *(ii)* *If α≥1, $X_{mRSS}^{\left(n\right)}\geq_{IG}X_{RSS}^{\left(n\right)}\geq_{IG}X_{MRSS}^{\left(n\right)}$.*

**Proof.**  From the DFR assumption of the underling distribution, it is known that

$$X_{1:n}\leq_{disp}X_{i:n}\leq_{disp}X_{(i)i},\;i=1,\ldots ,n;$$

see Shaked and Shantikumar (2007). Therefore, from Lemma 2 and for α≤1, we get

$$G_{\alpha }\left(X_{1:i}\right)\leq G_{\alpha }\left(X_{i:n}\right)\leq G_{\alpha }\left(X_{(i)i}\right),\;i=1,\ldots ,n,$$

and consequently,

$$\prod_{i=1}^{n}G_{\alpha }\left(X_{1:i}\right)\leq \prod_{i=1}^{n}G_{\alpha }\left(X_{i:n}\right)\leq \prod_{i=1}^{n}G_{\alpha }\left(X_{(i)i}\right).$$

Now, from the above inequality and definitions of the IG measures for vectors $X_{mRSS}^{\left(n\right)}$,$X_{RSS}^{\left(n\right)}$ and $X_{MRSS}^{\left(n\right)},$ we immediately obtain

$$G_{\alpha }\left(X_{mRSS}^{\left(n\right)}\right)\leq G_{\alpha }\left(X_{RSS}^{\left(n\right)}\right)\leq G_{\alpha }\left(X_{MRSS}^{\left(n\right)}\right),$$

which is equivalent to

$$X_{mRSS}^{\left(n\right)}\leq_{IG}X_{RSS}^{\left(n\right)}\leq_{IG}X_{MRSS}^{\left(n\right)},$$

which proves Part (i). Part (ii) can be proved in an analogous manner. □

**Theorem** **7.** *Let X and Y be independent random variables with densities f and g, respectively, and $X\leq_{disp}Y$. Then:*

 *(i)* *If α≤1, $X_{RSS}^{\left(n\right)}\leq_{IG}Y_{RSS}^{\left(n\right)}$ ;*
 *(ii)* *If α≥1, $Y_{RSS}^{\left(n\right)}\leq_{IG}X_{RSS}^{\left(n\right)}$.*

**Proof.**  By the definition of IG measure for RSS in (9), we have

$$G_{\alpha }\left(X_{RSS}^{\left(n\right)}\right)=\prod_{i=1}^{n}G_{\alpha }\left(X_{i:n}\right)=\psi \left(\alpha ,n\right)\prod_{i=1}^{n}E\left[f^{\alpha -1}\left(F^{-1}\left(V_{i}\right)\right)\right].$$

Because $X\leq_{disp}Y$, we have $f\left(F^{-1}\left(u\right)\right)\geq g\left(G^{-1}\left(u\right)\right)$ for all u∈(0,1), and so for α≤1, we get $f^{\alpha -1}\left(F^{-1}\left(u\right)\right)\leq g^{\alpha -1}\left(G^{-1}\left(u\right)\right).$ Now, making use of this inequality, we obtain

$$\begin{array}{ccc} G_{\alpha }\left(X_{RSS}^{\left(n\right)}\right) & = & \prod_{i=1}^{n}\frac{1}{B^{\alpha }\left(i,n-i+1\right)}\int_{0}^{1}u^{\alpha (i-1)}{\left(1-u\right)}^{\alpha (n-i)}f^{\alpha -1}\left(F^{-1}\left(u\right)\right)du\\ & \leq & \prod_{i=1}^{n}\frac{1}{B^{\alpha }\left(i,n-i+1\right)}\int_{0}^{1}u^{\alpha (i-1)}{\left(1-u\right)}^{\alpha (n-i)}g^{\alpha -1}\left(G^{-1}\left(u\right)\right)du=G_{\alpha }\left(Y_{RSS}^{\left(n\right)}\right), \end{array}$$

which proves Part (i). Part (ii) can be proved in an analogous manner. □

**Corollary** **1.** *Let X and Y be independent random variables with densities f and g, respectively, and $X\leq_{disp}Y$. Then:*

 *(i)* *If α≤1, $X_{mRSS}^{\left(n\right)}\leq_{IG}Y_{mRSS}^{\left(n\right)}$ ;*
 *(ii)* *If α≥1, $Y_{mRSS}^{\left(n\right)}\leq_{IG}X_{mRSS}^{\left(n\right)}$;*
 *(iii)* *If α≤1, $X_{MRSS}^{\left(n\right)}\leq_{IG}Y_{MRSS}^{\left(n\right)}$ ;*
 *(iv)* *If α≥1, $Y_{MRSS}^{\left(n\right)}\leq_{IG}X_{MRSS}^{\left(n\right)}.$*

## 4. RIG Divergence Measure Based on RSS Scheme

Let $X_{SRS}=\left\{X_{i},\;i=1,\ldots ,n\right\}$ denote a SRS of size *n* from density function (PDF) *f* and cumulative distribution function *F*. Further, let $X_{RSS}^{\left(n\right)}$, $X_{mRSS}^{\left(n\right)}$ and $X_{MRSS}^{\left(n\right)}$ be the corresponding RSS, minimum RSS and maximum RSS vectors, respectively. We now consider the RIG measure between variable *X* and each of the vectors $X_{mRSS}^{\left(n\right)}$ and $X_{MRSS}^{\left(n\right)}$. From the definition of RIG measure in (3), the RIG divergence between $X_{(1)i}$ with density in (6) and *X* is given by

$$\begin{array}{ccc} R_{\alpha }\left(X_{(1)i},X\right) & = & \int_{-\infty }^{\infty }f_{(1)i}^{\alpha }\left(x\right)f^{1-\alpha }\left(x\right)dx=i^{\alpha }\int_{0}^{1}{\left(1-u\right)}^{\alpha (i-1)}du=\frac{i^{\alpha }}{\alpha (i-1)+1}. \end{array}$$

Similarly, the RIG divergence between $X_{(i)i}$ with density in (7) and *X* is given by

$$\begin{array}{ccc} R_{\alpha }\left(X_{(i)i},X\right) & = & \int_{-\infty }^{\infty }f_{(i)i}^{\alpha }\left(x\right)f^{1-\alpha }\left(x\right)dx=i^{\alpha }\int_{0}^{1}u^{\alpha (i-1)}du=\frac{i^{\alpha }}{\alpha (i-1)+1}. \end{array}$$

It is evident from the above results that $R_{\alpha }\left(X_{(1)i},X\right)=R_{\alpha }\left(X_{(i)i},X\right),$ which is free of the underling distribution *F*.

**Theorem** **8.** *Consider the vectors $X_{SRS}^{\left(n\right)}$ and $X_{mRSS}^{\left(n\right)}$ from density function f. Then, we have:*

 *(i)* *$R_{\alpha }\left(X_{mRSS}^{\left(n\right)},X_{SRS}^{\left(n\right)}\right)=\prod_{i=1}^{n}R_{\alpha }\left(X_{(1)i},X\right)=c\left(\alpha ,n\right)$;*
 *(ii)*
$R_{\alpha }\left(X_{MRSS}^{\left(n\right)},X_{SRS}^{\left(n\right)}\right)=\prod_{i=1}^{n}R_{\alpha }\left(X_{(i)i},X\right)=c\left(\alpha ,n\right),$

*where $c\left(\alpha ,n\right)=\frac{{\left(n!\right)}^{\alpha }}{\prod_{i=1}^{n}\left(\alpha \left(i-1\right)+1\right)}.$*

**Proof.**  From the definition of RIG divergence between vectors $X_{SRS}^{n}$ and $X_{RSS}^{n}$, we find

$$\begin{array}{ccc} R_{\alpha }\left(X_{mRSS}^{\left(n\right)},X_{SRS}^{\left(n\right)}\right) & = & \int \cdots \int f_{(1)1}^{\alpha }\left(x_{1}\right)\cdots f_{(1)n}^{\alpha }\left(x_{n}\right)f^{1-\alpha }\left(x_{1}\right)\cdots f^{1-\alpha }\left(x_{n}\right)dx_{1}\cdots dx_{n}\\ & = & \prod_{i=1}^{n}\int f_{(1)i}^{\alpha }\left(x\right)f^{1-\alpha }\left(x\right)dx\\ & = & \prod_{i=1}^{n}R_{\alpha }\left(X_{(1)i},X\right)\\ & = & c(\alpha ,n), \end{array}$$

which proves Part (i). Part (ii) can be proved in an analogous manner. □

**Figure 2:**
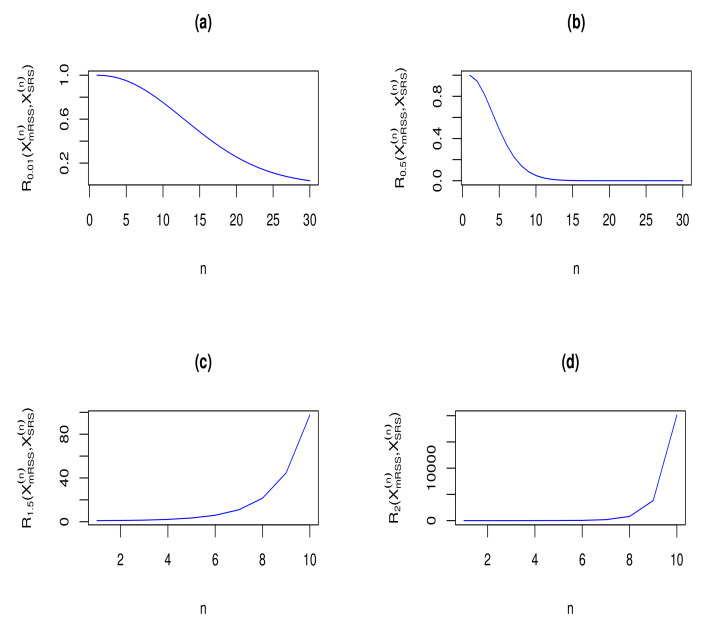
$R_{\alpha }\left(X_{mRSS}^{\left(n\right)},X_{SRS}^{\left(n\right)}\right)$ for some selected choices of parameter α and sample size *n*.

With the result that $R_{\alpha }\left(X_{mRSS}^{\left(n\right)},X_{SRS}^{\left(n\right)}\right)=R_{\alpha }\left(X_{MRSS}^{\left(n\right)},X_{SRS}^{\left(n\right)}\right)=\frac{{\left(n!\right)}^{\alpha }}{\prod_{i=1}^{n}\left(\alpha \left(i-1\right)+1\right)}$ in Theorem 8, we have plotted the RIG measure between vectors $X_{mRSS}^{\left(n\right)}$ and $X_{SRS}^{\left(n\right)}$, for some selected choices of α and sample size *n*, in Figure 2. From Figure 2, it is easy to observe that for α∈(0,1], the RIG divergence measure between $X_{mRSS}^{\left(n\right)}$ and $X_{SRS}^{\left(n\right)}$ is decreasing with respect to sample size *n* (Panels (a) and (b)), while for α∈[1,∞), the considered RIG measure is increasing with respect to sample size *n* (Panels (c) and (d)). Therefore, for α∈(0,1], the similarity between the density functions of the considered sampling vectors $X_{mRSS}^{\left(n\right)}$ and $X_{SRS}^{\left(n\right)}$ gets increased. For α∈[1,∞), the result is the opposite, i.e., the similarity between the two sampling vectors gets decreased.

**Theorem** **9.** *Consider the vectors $X_{RSS}^{\left(n\right)}$ and $X_{mRSS}^{\left(n\right)}$ from density function f. Then, we have:*

 *(i)* $R_{\alpha }\left(X_{mRSS}^{\left(n\right)},X_{RSS}^{\left(n\right)}\right)=\prod_{i=1}^{n}R_{\alpha }\left(X_{(1)i},X_{i:n}\right)=c^{*}\left(\alpha ,n\right)$;
 *(ii)*
$R_{\alpha }\left(X_{MRSS}^{\left(n\right)},X_{RSS}^{\left(n\right)}\right)=\prod_{i=1}^{n}R_{\alpha }\left(X_{(i)i},X_{(1)i}\right)=n!\prod_{i=1}^{n}\frac{\Gamma (\alpha (i-1)+1)\Gamma ((1-\alpha )(i-1)+1)}{\Gamma (i+1)},$

where c*(α,n)=n(n−1)!α∏i=1nn−1i−11−αΓ(α−i(α−1))Γ(α(2i−n−1)+n−i+1)Γ(α(i−n)+n+1).

**Proof.**  From the definition of RIG measure between vectors $X_{mRSS}^{\left(n\right)}$ and $X_{RSS}^{\left(n\right)}$, we have

Rα(XmRSS(n),XRSS(n))=∏i=1n∫f(1)iα(x)fi:n1−α(x)dx=(n!)αnα−1∏i=1n∫01n−1i−11−α(1−u)α(2i−n−1)+n−iu(1−α)(i−1)du=n(n−1)!α∏i=1nn−1i−11−αΓ(α−i(α−1))Γ(α(2i−n−1)+n−i+1)Γ(α(i−n)+n+1),

which proves Part (i). Part (ii) can be proved in a similar manner. □

**Figure 3:**
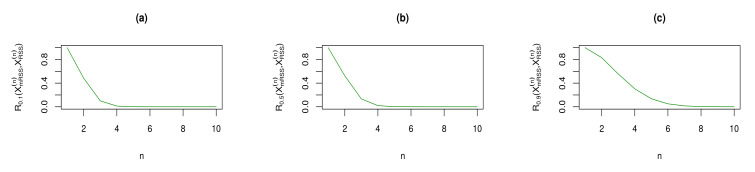
$R_{\alpha }\left(X_{mRSS}^{\left(n\right)},X_{RSS}^{\left(n\right)}\right)$ for some choices of parameter α and sample size *n*.

**Figure 4:**
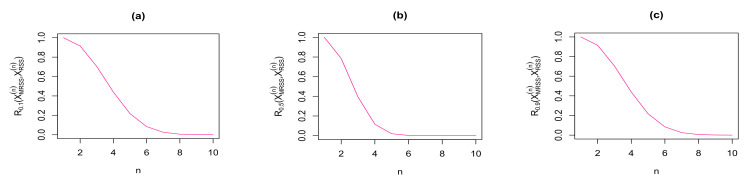
$R_{\alpha }\left(X_{MRSS}^{\left(n\right)},X_{RSS}^{n}\right)$ for some choices of parameter α and sample size *n*.

We have plotted the results of Theorem 9 in Figure 3 and Figure 4 for some choices of α. From these figures, we observe that for α∈(0,1], both RIG measures in Theorem 9 are deceasing with respect to sample size *n*. Therefore, the similarity between the density functions of the considered sampling vectors $X_{mRSS}^{\left(n\right)}$ and $X_{RSS}^{\left(n\right)}$ gets increased with increasing sample size *n*.

## 5. Concluding Remarks

In this paper, we have studied the information generating (IG) function and relative information generating (RIG) function measures associated with SRS and RSS strategies. Specifically, we have examined the IG function for maximum and minimum RSS schemes. We have shown that, under a mild condition on the density function *f*, for α≥1, the IG function associated with the sampling vector $X_{MRSS}^{\left(n\right)}$ is increasing with respect to sample size *n*. On the other hand, for α≤1, this function is decreasing. Similar results are established for the IG function of sampling vector $X_{mRSS}^{\left(n\right)}$ based on values of α and *n*. We have shown that for values of α≥1, we can provide upper bounds for $G_{\alpha }\left(X_{mRSS}^{\left(n\right)}\right)$ and $G_{\alpha }\left(X_{MRSS}^{\left(n\right)}\right)$ based on $G_{\alpha }\left(X_{SRS}^{\left(n\right)}\right)$. We have also provided some comparative results for RSS schemes in terms of dispersive stochastic ordering. Based on this stochastic ordering, we have established some ordering results among the IG functions of sampling vectors $X_{RSS}^{\left(n\right)}$, $X_{mRSS}^{\left(n\right)}$ and $X_{MRSS}^{\left(n\right)}$ in terms of α≥1 (or α≤1). Finally, we have examined the RIG measure between the vectors $X_{SRS}^{\left(n\right)}$, $X_{RSS}^{\left(n\right)}$, $X_{mRSS}^{\left(n\right)}$ and $X_{MRSS}^{\left(n\right)}$. The corresponding results associated with RIG divergence have been plotted in Figure 2, Figure 3 and Figure 4. For example, Figure 3 and Figure 4 present both RIG measures presented in Theorem 9 for some choices of α. We have demonstrated that the similarity between the density functions of the considered sampling vectors $X_{mRSS}^{\left(n\right)}$ and $X_{RSS}^{\left(n\right)}$ gets increased when the sample size *n* increases.

## Data Availability

Data sharing not applicable.
